# Social patterning of acute respiratory illnesses in the Household Influenza Vaccine Evaluation (HIVE) Study 2014–2015

**DOI:** 10.1017/S0950268819000748

**Published:** 2019-05-02

**Authors:** Ryan E. Malosh, Grace A. Noppert, Jon Zelner, Emily T. Martin, Arnold S. Monto

**Affiliations:** 1 University of Michigan School of Public Health, Ann Arbor, MI, USA; 2 Carolina Population Center, University of North Carolina, Chapel Hill, NC, USA

**Keywords:** Epidemiology, respiratory infections, social determinants of health

## Abstract

Social patterning of infectious diseases is increasingly recognised. Previous studies of social determinants of acute respiratory illness (ARI) have found that highly educated and lower income families experience more illnesses. Subjective social status (SSS) has also been linked to symptomatic ARI, but the association may be confounded by household composition. We examined SSS and ARI in the Household Influenza Vaccine Evaluation (HIVE) Study in 2014–2015. We used SSS as a marker of social disadvantage and created a workplace disadvantage score for working adults. We examined the association between these measures and ARI incidence using mixed-effects Poisson regression models with random intercepts to account for household clustering. In univariate analyses, mean ARI was higher among children <5 years old (*P* < 0.001), and females (*P* = 0.004) at the individual level. At the household level, mean ARI was higher for households with at least one child <5 years than for those without (*P* = 0.002). In adjusted models, individuals in the lowest tertile of SSS had borderline significantly higher rates of ARI than those in the highest tertile (incidence rate ratio (IRR) 1.34, 95% confidence interval (CI) 0.98–1.92). Households in the lowest tertile of SSS had significantly higher ARI incidence in household-level models (IRR 1.46, 95% CI 1.05–2.03). We observed no association between workplace disadvantage and ARI. We detected an increase in the incidence of ARI for households with low SSS compared with those with high SSS, suggesting that socio-economic position has a meaningful impact on ARI incidence.

## Introduction

Social disadvantage shapes the distribution of a wide range of health outcomes, including cardiovascular disease [1], cancer incidence and all-cause mortality [2]. The impact of social disadvantage on health is frequently captured using indicators of socio-economic position (SEP) such as income, education and occupation [3], and related measures such as subjective social status (SSS) [4]. Estimating the effects of social disadvantage is challenging because no single measure can fully capture the multi-dimensional relationship between SEP and health, and these measures may not be interchangeable across populations, cohorts or the life course [5].

These issues are particularly pronounced for infectious disease researchers attempting to utilise the measures of social disadvantage. Surveillance data, for example, are typically lacking individual-level data necessary to capture the impact of social disadvantage on infection outcomes while controlling for potential confounders such as age and household composition. Thus, infectious disease researchers responding to the call for a greater incorporation of social measures into their studies [6, 7] are faced with both data and measurement challenges. Nevertheless, consistent positive associations between social disadvantage and risk have been observed with certain infectious diseases such as human immunodeficiency virus [8], tuberculosis [9–11] and pandemic influenza [12]. More recently, social disadvantage has been shown to be a risk factor for chronic infections with pathogens such as cytomegalovirus, herpes simplex virus type-1, *Helicobacter pylori* (*H. pylori*) and *Chlamydia pneumonia* (*C. pneumoniae*) [13–18].

There are several mechanisms by which social disadvantage may be linked to acute illnesses. Common social epidemiologic frameworks suggest that individuals experiencing increased levels of social disadvantage may be more likely to live and work in environments where they may be more exposed to infections, with fewer resources available to cope with these infections. However, because social factors have long been considered an unimportant part of the ecology of acute infections, research into the social determinants of susceptibility to acute infections such as seasonal influenza is less common and often focuses on access to preventive interventions rather than upstream causes [19].

The purpose of the current study was to test whether social disadvantage could predict the incidence of acute respiratory illness (ARI). We used data collected from the Household Influenza Vaccine Evaluation (HIVE) Study designed to evaluate vaccine effectiveness and examine influenza transmission in households. We hypothesised that increasing social disadvantage, at both the individual- and household-level, would be associated with increased incidence of ARI.

## Methods

### Participants

This study is a secondary analysis of data collected during the 2014–2015 season of the HIVE study. The HIVE study, based on the landmark Tecumseh Study of Respiratory Illness [20], is an ongoing, prospective cohort study of households with children in and around Ann Arbor, MI. Eligible households with ⩾3 members, including ⩾2 children <18 years, were identified, recruited and enrolled from June through September 2014 and followed for incident ARI from October 2014 through May 2015, as previously described [21]. Adult household members provided written informed consent for participation for themselves and their children; children 7–17 years provided oral assent. All study visits were carried out at the University of Michigan School of Public Health (UM-SPH). Surveys were administered using online survey software (Qualtrics; Provo, UT). The University of Michigan Medical School institutional review board reviewed and approved the study.

### Predictor variables

We explored social and workplace disadvantage at both the individual and household level. We used SSS as a proxy measure of social disadvantage. At enrolment, adult household members reported household SSS, using a nine-point ladder question adapted from the MacArthur scale of SSS [22]. This value was assigned to each household member. Prior to analysis, we examined the distribution of SSS and categorised individuals and households as above, at or below the median value of SSS.

Workplace disadvantage was measured using individual responses to a series of questions regarding the work environment. Adult household members were queried about work outside the home, and working adults were asked to state their level of agreement (on a five-point Likert scale) with three items that characterise workplace-related acute illness policies and exposure risks. These items were: (1) Employees are discouraged from coming to work when they have flu symptoms, (2) Employees are encouraged to go home if they have flu symptoms at work and (3) I have a lot of control over when I can schedule days off from work for illnesses or doctor appointments (Table S1). Importantly, workplace sick leave policies have been shown to be a critical component of SES and an important factor affecting differential exposure to pathogens [23]. We aggregated the responses to these questions to create a composite workplace disadvantage score which was categorised in quartiles. Only working adult respondents were included in individual-level models of workplace disadvantage.

For household-level analyses, we used reported household-level SSS. We also averaged all of the working adult respondent scores in each household to create an average household workplace disadvantage score, which was then categorised into quartiles. We believe the household-level analyses are complementary to the individual-level analyses. It allowed us to explore additional predictors of ARI (e.g. number of children and household size).

### Outcome

The primary outcome of interest was the seasonal incidence rate of ARI. ARI surveillance was carried out from October 2014 through May 2015. Households were instructed to report all ARI at illness onset and were queried weekly to identify newly onset ARI. Case definitions for eligible illnesses were defined by symptoms tailored to individuals ⩾3 years of age and children <3 years of age. For individuals ⩾3 years of age, incident ARI was defined by reporting two or more of the following symptoms: *cough*, *fever/feverishness*, *nasal congestion*, *chills*, *headache*, *body aches or sore throat.* For children <3 years of age, incident ARI was defined by reporting two or more of the following symptoms: *cough*, *fever/feverishness*, *runny nose/congestion*, *difficulty breathing*, *fussiness/irritability*, *fatigue or loss of appetite*.

### Additional variables

At enrolment, study participants reported demographic characteristics and health history (e.g. comorbid conditions). They also reported the total number of individuals living in their home as well as whether each individual worked outside the home or attended school or childcare. Participants >16 years old were also asked to report how often they smoke cigarettes (not at all, some days, every day). Given that the survey was administered upon enrolment in the study, there were no missing values on either SSS or covariates.

### Statistical analysis

We first described the SSS and workplace disadvantage score by both individual and household characteristics using Student's *t* tests to compare the mean score for dichotomous variables and ANOVA models for variables with multiple categories. We then plotted distributions of the number of ARI per individual by age category, and sex. We also plotted the distribution of ARI per household by total number of people and number of children <5 years old.

We estimated the association between SSS and workplace disadvantage using Poisson regression models. Individual- and household-level models were run separately. At the individual-level, we used mixed-effects Poisson models including a random intercept for household to account for correlations in ARI reporting and SSS between household members [24]. We report the fixed-effect estimates from these models as an estimate of the incidence rate ratio (IRR) for each covariate. The percentile method was used to construct bootstrap confidence intervals for each of these fixed-effect estimates using 1000 resampling frames [25]. Household-level effects of SSS and workplace disadvantage on ARI incidence were estimated using negative binomial models for count data.

All analyses were conducted in R version 3.4.3. IRR and 95% confidence intervals (CIs) were estimated using the *lmer4* package. A two-sided *P*-value of 0.05 was used to determine statistical significance.

## Results

### Study population characteristics

In total, 1431 individuals from 340 households participated in the HIVE study during the 2014–2015 season. Overall, 60% of the study population were children <18 years old and 36% were adults 18–49 years old. SSS ranged from 1 to 9 and the median was 7 (IQR 6–7). The age distribution of households differed by SSS category (*P* = 0.01), but no differences were observed in the proportion of males or in the proportion working or attending school or childcare outside the home (Table 1).

**Table 1.** Number and proportion^a^ of individuals by tertile of subjective social status and quartile of workplace disadvantage score

	Subjective social status (*N* = 1431 individuals)					Workplace disadvantage score (*N* = 384 working adults)					
	Total	>7	7	<7	*P*-value^b^	Total	<4	4–5	6–7	>7	*P*-value^b^
Individuals	1431	334	557	540		384	68	107	121	88	
Age category					0.01						0.18
0–4	200 (14)	48 (14)	64 (11)	88 (16)		–	–	–	–	–	
5–11	442 (31)	98 (29)	163 (29)	181 (34)		–	–	–	–	–	
12–17	220 (15)	49 (15)	92 (17)	74 (14)		–	–	–	–	–	
18–49	508 (36)	123 (37)	204 (37)	186 (34)		348 (91)	62 (91)	101 (94)	104 (86)	81 (92)	
50+	61 (4)	16 (5)	34 (6)	11 (2)		36 (9)	6 (9)	6 (6)	17 (14)	7 (8)	
Sex					0.04						0.39
Female	746 (52)	155 (46)	294 (53)	297 (55)		211 (55)	41 (60)	60 (56)	59 (49)	51 (58)	
Male	685 (48)	179 (54)	263 (47)	243 (45)		173 (45)	27 (40)	47 (44)	62 (51)	37 (42)	
Work/school/daycare					0.25						
Yes	1221 (85)	282 (84)	486 (87)	453 (84)		–	–	–	–	–	
No	210 (15)	52 (16)	71 (13)	87 (16)		–	–	–	–	–	

a The per cent of the column total

b *P*-value from *χ*^2^ test, or Fisher's exact test when individual cell sizes are less than *n* = 10.

Three hundred and eighty-four working adults responded to the survey and answered questions about their workplace environment. The workplace disadvantage score ranged from 1 to 15 and the median was 6 (IQR 4–7). Of the 340 households participating in the study, 262 (86%) included at least one working adult who responded to the workplace disadvantage questions. The majority of the households had four members (range 3–9), households in the lowest tertile of SSS were disproportionately large (*P* = 0.02) compared with those with higher SSS (i.e. greater proportion of households with ⩾5 individuals). The majority of households (58%) had no children under 5 years of age (Table 2).

**Table 2.** Number and proportion^a^ of households by tertile of subjective social status and quartile of workplace disadvantage score

	Subjective social status (*N* = 340 households)					Workplace disadvantage score (*N* = 262 households)					
	*N* (%)	>7	7	<7	*P*-value^b^	*N* (%)	<4	4–5	5–6	⩾7	*P*-value^b^
Households	340	79	133	128		262	54	73	76	59	
Household size					0.02						0.20
3	64 (19)	11 (14)	19 (14)	34 (27)		39 (15)	10 (19)	6 (8)	11 (14)	12 (20)	
4	186 (55)	48 (61)	82 (32)	56 (44)		151 (58)	34 (63)	47 (64)	39 (51)	31 (53)	
5+	90 (26)	20 (25)	32 (24)	38 (30)		70 (27)	10 (19)	20 (27)	26 (34)	16 (27)	
Children <5 years old in household					0.31						0.94
0	196 (58)	44 (56)	83 (62)	69 (54)		158 (60)	33 (61)	44 (60)	48 (63)	33 (56)	
1	94 (28)	24 (30)	36 (27)	34 (27)		64 (24)	14 (26)	19 (26)	17 (22)	14 (24)	
2+	50 (15)	11 (14)	14 (11)	25 (20)		40 (15)	7 (13)	10 (13)	11 (14)	12 (20)	

a The per cent of the column total.

b *P*-value from *χ*^2^ test, or Fisher's exact test when individual cell sizes are less than *n* = 10.

### Distribution of ARI by individual- and household-level characteristics

**Fig. 1.**
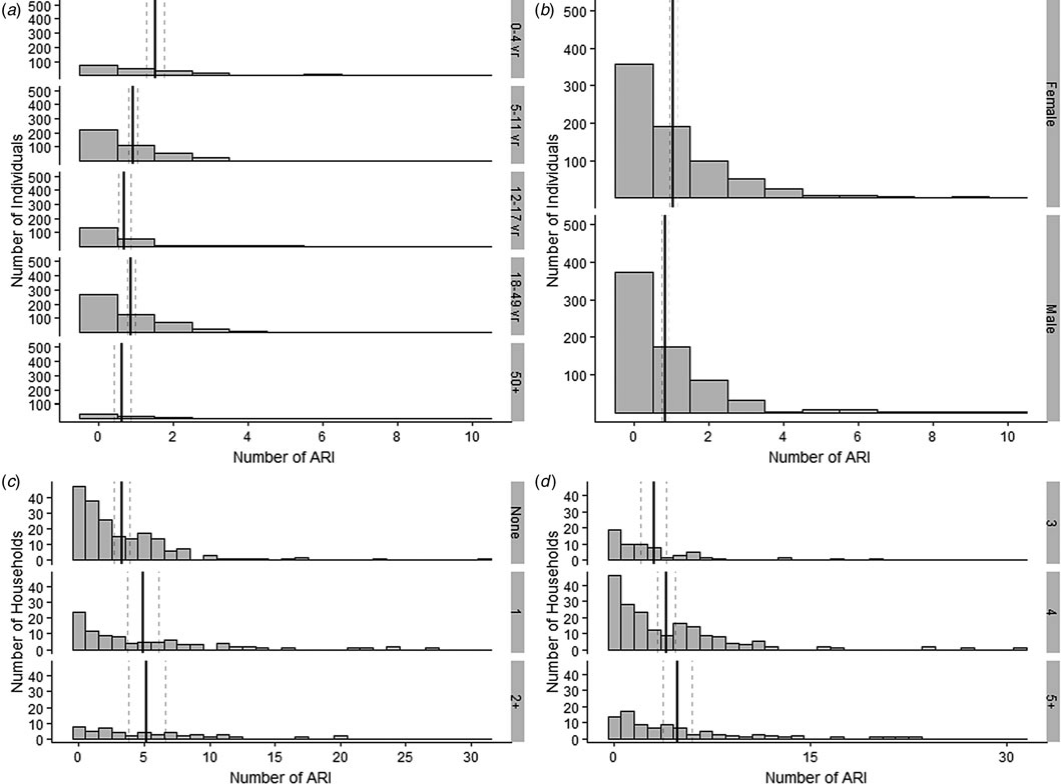
Distribution of ARI by individual and household characteristics. Solid vertical lines represent group mean, dashed lines represent 95% CI around the mean ARI: (a) distribution of individuals by number of ARI reported, stratified by age category; (b) distribution of individuals by number of ARI reported, stratified by gender; (c) distribution of households by number of ARI reported, stratified by number of children <5 years living in the household; (d) distribution of households by number of ARI reported, stratified by total household size.

Overall there were 1362 ARI reported among 1431 individuals in the study (mean # of ARI events = 0.95, 95% CI 0.88–1.02). Figure 1 presents the distribution of ARI by individual- and household-level factors. Children 0–4 years old had the highest frequency of ARI (mean 1.53, 95% CI 1.28–1.78), followed by children 5–11 years old (mean 0.94, 95% CI 0.81–1.06) and adults 18–49 years old (mean 0.88, 95% CI 0.77–0.99). Females reported more ARI than males.

Households with no children <5 years old reported fewer ARIs (mean 3.28, 95% CI 2.70–3.85) than those with at least one child in this age group; however, there did not appear to be a linear trend as households with one child <5 years (mean 4.89, 95% CI 3.68–6.10) reported similar frequency of ARI as households with two or more children <5 years (5.2, 95% CI 3.82–6.58). Larger households also reported more ARI than those with fewer members.

### SSS and ARI

**Fig. 2.**
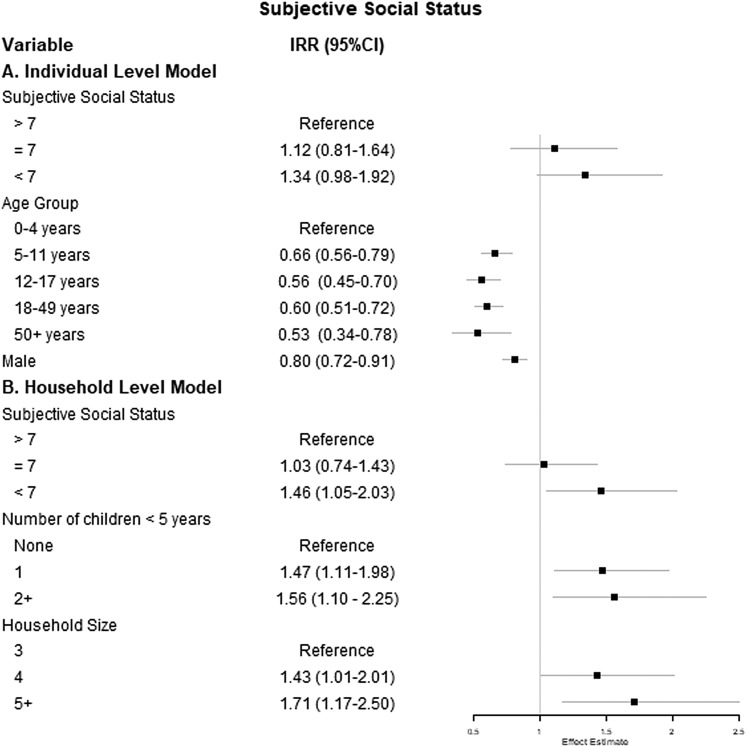
Results of (a) individual-level multivariable mixed-effects and (b) household-level multivariable count models examining the association between subjective social status and count of ARI. Note: The individual-level model is adjusted for age group, sex and working or attending school or childcare outside the home. The household-level model is adjusted for number of children <5 years of age and household size.

At the individual level, those below the median SSS (<7) had a borderline significantly higher incidence of ARI compared with those above the median (>7) in a multivariable mixed-effects Poisson regression model (Fig. 2a). Controlling for age group, sex and working or attending school or childcare outside the home, individuals below the median SSS had a 34% increase in the incidence rate of ARI, compared with those above the median SSS (IRR 1.34, 95% CI 0.98–1.92). The 95% CI for this estimate included the null value, indicating that the finding is not statistically significant. Similarly, individuals reporting median levels of SSS had 12% higher incidence of ARI than those reporting the highest levels of SSS (IRR 1.12, 95% CI 0.81–1.64), but this finding was also not statistically significant. Age group was significantly associated with the incidence of ARI; children 0–4 years had the highest incidence. Males also had a lower incidence of ARI than females (IRR 0.80, 95% CI 0.72–0.91).

In household-level models, we found a significantly higher incidence of ARI for those with low SSS. Households reporting SSS below the median had a 46% increase in the incidence of ARI (IRR 1.46, 95% CI 1.05–2.03) compared with households above the median (Fig. 2b). The number of children <5 years of age and household size were also significant predictors of ARI incidence in a household-level model. Compared with households with no children <5 years old, those with one child in this age group had 47% higher incidence of ARI (IRR 1.47, 95% CI 1.11–1.98); similar results were found for those with two or more children <5 years old (IRR 1.56, 95% CI 1.10–2.25).

### Workplace disadvantage and ARI

**Fig. 3.**
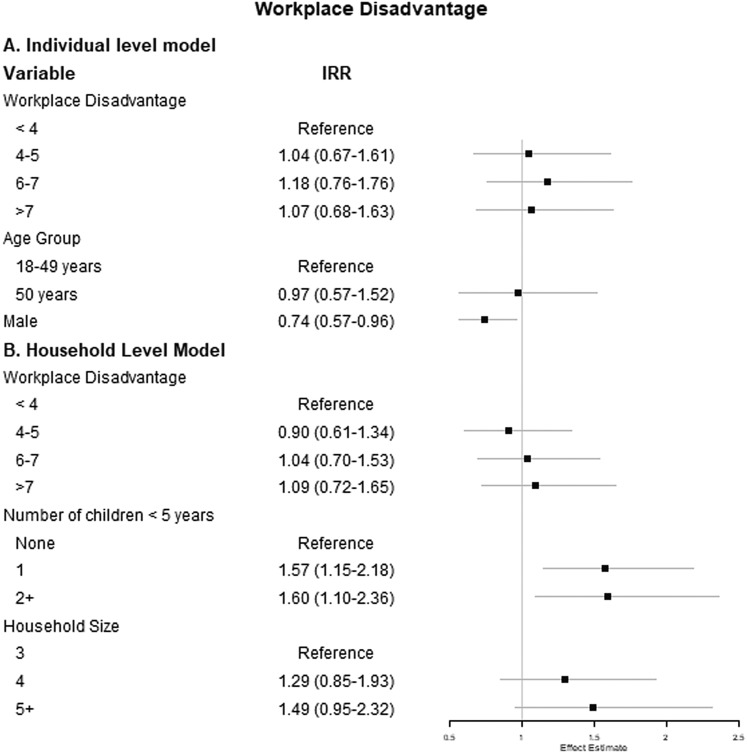
Results of (a) individual-level multivariable mixed-effects and (b) household-level multivariable count models examining the association between workplace disadvantage score and count of ARI. Note: The individual-level model is adjusted for age group, sex and working or attending school or childcare outside the home. The household-level model is adjusted for number of children <5 years of age and household size.

We also evaluated the associations between workplace disadvantage and incident ARI separately at the individual- and household-level. In a multivariable count model predicting ARI incidence, the only significant predictor of ARI incidence among working adults was sex (Fig. 3a), with men having lower incidence compared with women (IRR 0.74, 95% CI 0.57–0.96).

At the household level, the number of children <5 years of age was the only significant predictor of the incidence of ARI (Fig. 3b). Specifically, having two or more children <5 years of age in the household was associated with a 60% increase in the IRR of ARI compared with households with no children <5 years of age, though this finding was only borderline statistically significant (IRR 1.60, 95% CI 1.10–2.36).

## Discussion

We used a community-based cohort study to examine whether ARI risk reflected social stratification among individuals and households. We found higher levels of social disadvantage, as measured by lower SSS, were associated with increasing incidence of ARI at the household-level. We also found a non-significant increase in ARI incidence for individuals with lower SSS. Workplace disadvantage, however, was not associated with ARI in either individual or household models. Our findings demonstrate that social stratification was detectable even among common acute illnesses such as ARI, and within a population that is not characterised by extreme disadvantage.

Evidence of social stratification in common illnesses has been intermittently reported for the last several decades. In a 1974 paper, Monto and Ullman reported the surprising finding that respiratory infection rates increased as the level of education of the head of household increased in the classic Tecumseh Study of Respiratory Illness [20]. Many studies have since reported differences in chronic infection rates by income, education and other markers of SEP [8, 10–12, 14, 26–28]. However, until the last decade, few others have examined subjective markers of social disadvantage. In a viral challenge study, Cohen *et al.* found that individuals reporting lower SSS (i.e. those with higher levels of disadvantage) at baseline were more likely to develop symptomatic illness, independent of traditional markers of social disadvantage [29]. In a follow-up study, SSS was found to be a key moderator of the impact of sleep duration on common cold infection [30]. Thompson *et al.* further tested this finding in a sample of health care workers and demonstrated, consistent with our results, that low SSS at baseline was associated with increased rates of ARI [31]. Together, these findings suggest that SSS, regardless of educational attainment and income, may be a key predictor of symptomatic illness. Importantly, these studies were unable to examine if the effects persisted after controlling for household composition.

Our findings demonstrated an association between increasing social disadvantage and increasing incidence of ARI. Two separate hypotheses could explain this observation: increased exposure to infection and biologic vulnerability. *Vis-à-vis* the first hypothesis, increasing social disadvantage would be associated with increased exposure to pathogens that cause ARI, thus resulting in an increased incidence of illness. Increased exposure to pathogens could be the result of poor housing conditions, lack of access to material resources and neighbourhood environments that limit access to healthcare. For example, poor housing conditions may include crowded living conditions in which individuals are living in close contact with others and thus are more likely to be exposed to a pathogen. Lack of material resources and/or the neighbourhood environment may limit one's ability to receive vaccination for certain viruses. This represents a neo-materialist approach to this pathway; though the relative homogeneity of the sample precludes a more thorough investigation of this theory [32]. The second hypothesis, on the other hand, posits that increasing social disadvantage would result in increased physiological wear and tear through mechanisms such as chronic stress. Thus, more socially disadvantaged individuals would be more likely to develop symptomatic infections when exposed to pathogens. Studies have found that chronic stress (related to low SES) is associated with increased inflammation [33, 34] and changes in immune function [35, 36], specifically cell-mediated immune function. New infections require activation of the naïve T-cell pool, increasing the numbers of memory T-cells and reducing naïve T-cells able to combat future infections. These changes could result in increased biologic vulnerability to infections both in the short-term and the long-term.

The studies by Cohen *et al.* on SSS seem to point to the plausibility of the biologic vulnerability hypothesis given the prospective nature of the studies and the uniform exposure of the viral challenge [29, 30]. We did not collect data on asymptomatic or sub-clinical infections and therefore could not explicitly replicate their findings in a real-world scenario. Nevertheless, the observation from this analysis that the incidence of symptomatic ARI increases at both the household- and individual-level with increasing social disadvantage lends some support to the original observation.

Extending the biologic vulnerability pathway, one could hypothesise that there is likely a long-term biological cost to repeated exposure to infection, even common infections such as ARI. This hypothesis may be a helpful explanation, not just for explaining ARI disparities, but for other more serious outcomes as well. Short-term infections, such as the common cold, could then be one mechanism by which prolonged exposure to social disadvantage may lead to poorer health outcomes overall. Repeated exposure to short-term processes such as the common cold may have long-term consequences for immune function and health, a process increasingly detrimental as individuals' age.

An alternative explanation of these results is that lower SSS increases the risk of certain behaviours that subsequently increase the risk of ARI. To examine one potential behavioural pathway, we explored smoking as a mediator of the association between SSS and ARI incidence among adult HIVE participants (Tables S3 and S4). In a causal mediation analysis, we found no evidence that smoking mediates the association between SSS and ARI at the individual level (Fig. S1). Another pathway we did not explore in this analysis was the potential reduction in ARI incidence by influenza vaccination. During the 2014–2015 season, the influenza vaccine was not effective in preventing influenza infections due to antigenic drift in the predominant circulating virus, influenza A/H3N2. Thus, no reduction in ARI incidence would be expected due to vaccination in the current analysis. Further, in years when the vaccine is effective, we believe that influenza vaccination may mediate the association between SSS and ARI. A rigorous analysis of these potential mediation effects will require additional seasons of influenza and ARI data and a modelling strategy adapted to this question [37]. Additionally, future studies would benefit from the inclusion of other markers of social status (e.g. education and income).

We did not observe an association between workplace disadvantage and the incidence of ARI. However, we cannot rule out that this type of disadvantage may manifest at other points in the infectious process (e.g. severity of infection or duration of infection) rather than simply influencing the incidence of infection. Measuring the effect of workplace disadvantage on acute infectious diseases will require more nuanced outcome measures. However, we believe that understanding the impact of workplace disadvantage, and specifically sick leave policies, is critical to addressing health disparities in both exposure to and incidence of ARI [23, 38].

We used the Household Influenza Vaccine Evaluation (HIVE) Study cohort to examine the association between social disadvantage and acute illnesses. By traditional metrics, including education and insurance coverage, our study population was not characterised by extreme variations in SEP [39]. Our study population may not be generalisable to more urban or rural populations; however, it is generalisable to many suburban communities, which make up the majority of the US population. These populations are typically not the focus of research studies examining social stratification and its consequences. Nevertheless, even in a population with limited variability in traditional markers of SEP, we were able to show the detrimental impact of social stratification on health. Additionally, it is possible that there was under-reporting of illnesses in this population. However, we believe this would result in an underestimation of the true effect size of the association between SSS and ARI.

## Supplementary material

For supplementary material accompanying this paper visit https://doi.org/10.1017/S0950268819000748. click here to view supplementary material
