# Supplementary material for: Social patterning of acute respiratory illnesses in the Household Influenza Vaccine Evaluation (HIVE) Study 2014–2015
Source: Epidemiol Infect. 2019 May 2;147:e185. doi: 10.1017/S0950268819000748 (PMC6518597; doi:10.1017/S0950268819000748)
Supplement: Supplementary file 1 [file S0950268819000748sup001.docx]

**Epidemiology and Infection**

**Title:** Social patterning of acute respiratory infections in the Household Influenza Vaccine Evaluation (HIVE) Study 2014-2015

**Authors:** RE Malosh, GA Noppert, J Zelner, ET Martin, AS Monto

**Supplementary Material**

*Workplace disadvantage score*

We queried adult household member about their work outside the home. Working adults were further questioned about their workplace environment with regard to acute respiratory illnesses. These questions were originally adapted from a study demonstrating that control over time off reduced the negative effects of workplace stress on absenteeism (1). The three items asked respondents to rate their agreement with statements about their workplace environment regarding policies about acute respiratory illnesses (or flu-like symptoms). The three items and their mean and standard deviation are listed in Table S1.

Table S1. Items comprising the workplace disadvantage score

| Item | Wording | Scale | Mean (SD) Response |
| --- | --- | --- | --- |
| 1 | Employees are discouraged from coming to work when they have flu symptoms | 5 point scale: Strongly Agree (1) – Strongly Disagree (5) | 2.01 (0.96) |
| 2 | Employees are encouraged to go home if they have flu symptoms at work | 5 point scale: Strongly Agree (1) – Strongly Disagree (5) | 2.03 (0.95) |
| 3 | I have a lot of control over when I can schedule days off from work for illnesses or doctor appointments | 5 point scale: Strongly Agree (1) – Strongly Disagree (5) | 1.98 (1.17) |

*Smoking as a mediator*

We conducted a causal mediation analysis using the R package *mediate* to estimate the Average Causal Mediation Effect (ACME). This function does not accept input from mixed effects Poisson regression models. Thus, we fit a multivariable Poisson regression model estimating the association between smoking status and ARI incidence, adjusting for potential confounders (age, sex, high risk conditions, work outside the home, and influenza vaccination status) and including an interaction term for smoking and SSS.

Table S2. Association between smoking and ARI, adjusted for potential confounders among adults (n=567) in the Household Influenza Vaccine Effectiveness (HIVE) Study 2014-2015

| **Covariate** | **IRR** | **95% CI** | **p-value** |
| --- | --- | --- | --- |
| Smoking | 0.65 | 0.07-6.70 | 0.71 |
| SSS | 1.08 | 1.01-1.15 | 0.03 |
| Male | 0.66 | 0.54-0.81 | < 0.001 |
| Age Category |  |  |  |
| 18-49 (reference) | -- | -- | -- |
| 50+ | 0.74 | 0.53-1.02 | 0.08 |
| Work/School outside home | 0.82 | 0.97-1.00 | 0.07 |
| Influenza vaccination | 1.10 | 0.98-1.20 | 0.07 |
| High-risk condition | 1.42 | 1.31-2.23 | 0.001 |
| SSS*Smoking | 1.10 | 0.72-1.57 | 0.63 |

Next, we fit a separate multivariable logistic regression model estimating the association between smoking status and SSS, adjusted for sex, and age category. We find that a one-unit increase in SSS is associated with 2.25 times the odds of smoking.

Table S3. Association between SSS and smoking, adjusted for sex among adults (n=567) in the Household Influenza Vaccine Effectiveness (HIVE) Study 2014-2015

| **Covariate** | **OR** | **95% CI** | **p-value** |
| --- | --- | --- | --- |
| SSS | 2.25 | 1.5-3.39 | <0.001 |
| Male | 1.31 | 0.26-5.66 | 0.73 |

Finally, we estimated the ACME, direct and total effects along with 95% confidence intervals. The method we used to estimate the ACME, developed my Imai and colleagues, is a comprehensive framework to identify and estimate causal mediation effects. To estimate the ACME of smoking on SSS we modeled SSS as a linear, continuous predictor variable. We then examined the ACME by specifying a contrast of interest, in this case an SSS of 4 (more disadvantaged than the median) was compared to an SSS of 2 (less disadvantaged than the median).


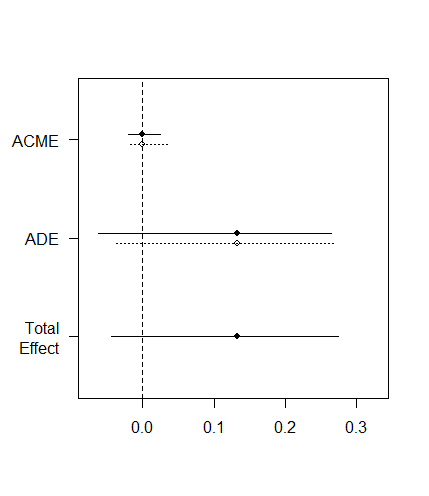


Figure S1. Estimates and 95% confidence intervals for the average causal mediation effect (ACME), average direct effect (ADE), and total effect examining smoking status as a mediator of the association between SSS and ARI.

We observe no causal mediation effect. The results of this analysis in conjunction with the absence of a significant effect of smoking on ARI from the Poisson models described above (Table S3) suggest that there is no evidence that smoking mediates the association between SSS and ARI at the individual level.
